# Characterisation of insulin analogues therapeutically available to patients

**DOI:** 10.1371/journal.pone.0195010

**Published:** 2018-03-29

**Authors:** Gary G. Adams, Andrew Meal, Paul S. Morgan, Qushmua E. Alzahrani, Hanne Zobel, Ryan Lithgo, M. Samil Kok, David T. M. Besong, Shahwar I. Jiwani, Simon Ballance, Stephen E. Harding, Naomi Chayen, Richard B. Gillis

**Affiliations:** 1 Faculty of Medicine and Health Sciences, University of Nottingham, Queens Medical Centre, Nottingham, NG7 2UH, United Kingdom; 2 National Centre for Macromolecular Hydrodynamics, University of Nottingham, School of Biosciences, Sutton Bonington, LE12 5RD, United Kingdom; 3 Taif University, Faculty of Science, Taif, Saudi Arabia; 4 Nofima AS, Osloveien 1, Ås, Norway; 5 Department of Food Engineering, Abant Izzet Baysal University, Bolu, Turkey; 6 Functional Nanomaterials Lab, King Abdullah University of Science and Technology (KAUST), Thuwal, Kingdom of Saudi Arabia; 7 Computational and Systems Medicine, Department of Surgery and Cancer, Faculty of Medicine, Imperial College London, London, SW7 2AZ, United Kingdom; Weizmann Institute of Science, ISRAEL

## Abstract

The structure and function of clinical dosage insulin and its analogues were assessed. This included ‘native insulins’ (human recombinant, bovine, porcine), ‘fast-acting analogues’ (aspart, glulisine, lispro) and ‘slow-acting analogues’ (glargine, detemir, degludec). Analytical ultracentrifugation, both sedimentation velocity and equilibrium experiments, were employed to yield distributions of both molar mass and sedimentation coefficient of all nine insulins. Size exclusion chromatography, coupled to multi-angle light scattering, was also used to explore the function of these analogues. On ultracentrifugation analysis, the insulins under investigation were found to be in numerous conformational states, however the majority of insulins were present in a primarily hexameric conformation. This was true for all native insulins and two fast-acting analogues. However, glargine was present as a dimer, detemir was a multi-hexameric system, degludec was a dodecamer (di-hexamer) and glulisine was present as a dimer-hexamer-dihexamer system. However, size-exclusion chromatography showed that the two hexameric fast-acting analogues (aspart and lispro) dissociated into monomers and dimers due to the lack of zinc in the mobile phase. This comprehensive study is the first time all nine insulins have been characterised in this way, the first time that insulin detemir have been studied using analytical ultracentrifugation and the first time that insulins aspart and glulisine have been studied using sedimentation equilibrium. The structure and function of these clinically administered insulins is of critical importance and this research adds novel data to an otherwise complex functional physiological protein.

## Introduction

A fundamental intention of insulin therapy is to mimic ‘normal’ physiological insulin secretion patterns, thus controlling basal and mealtime plasma glucose and fatty acid turnover optimising blood glucose control [1]. Under physiological conditions, prandial insulin secretion, which begins rapidly in response to autonomic activity and incretins, continuing with the rise in glucose and amino acid concentrations, is modulated on a 3–10 min timeframe; this is impossible to imitate without intravascular glucose sensing and insulin delivery [2].

In Diabetes Mellitus (DM), normal physiological processes do not occur and chronic hyperglycaemia is observable, where glycaemic control is severely impaired because of prevailing deficiencies in insulin or its action [3]. This insufficiency precipitates metabolic and degenerative microvascular and macrovascular complications in multiple organs including the heart, nerves, eyes and kidneys. Historically, bovine and porcine insulins were used to treat patients presenting with diabetes mellitus. Presently, insulins used at meal-times are associated with a lag-phase post-injection before absorption begins, a delay to peak because of tissue diffusion and long-acting insulins could potentially impact on physiological function initiating aberrant cell cycle progression [4,5]. In addition, mean glucose control remains poor in clinical practice, the challenge of hypoglycaemia continues to be problematic and user convenience still prevails.

**Fig 1:**
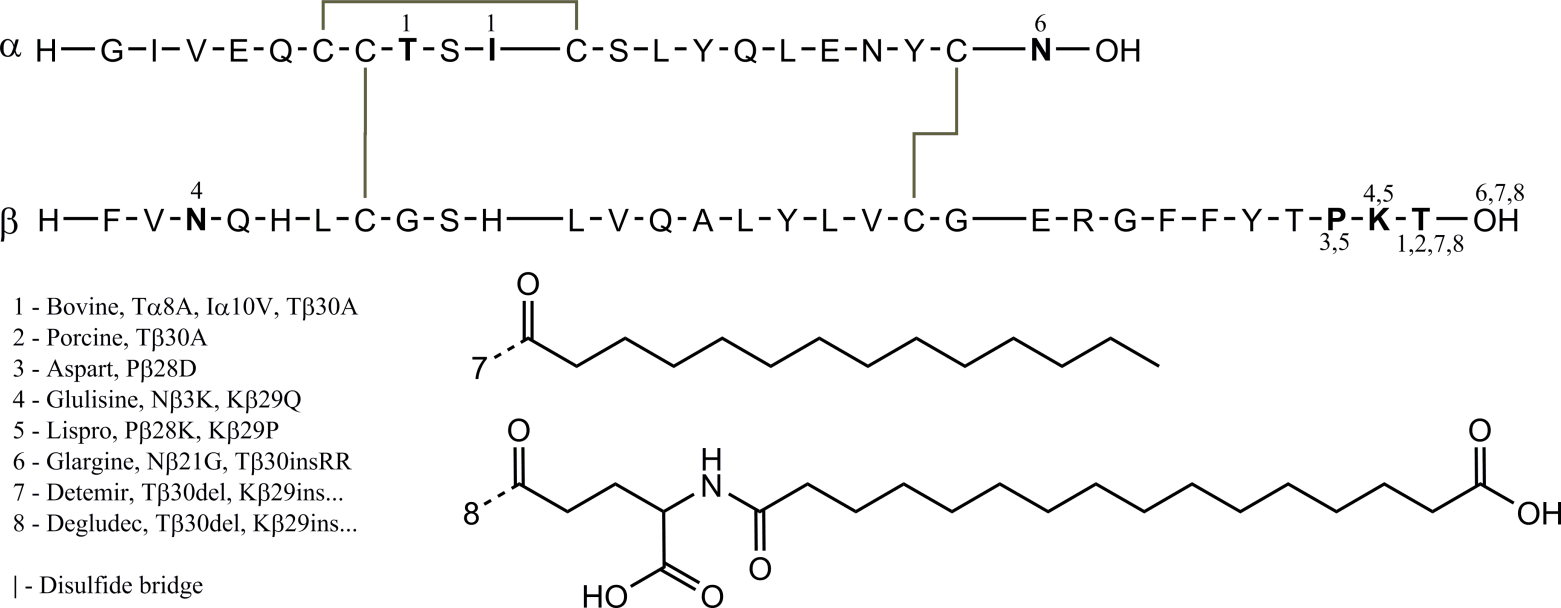
Primary structure of human insulin and its analogues. Differences highlighted and numbered.

Insulin is a hormonal protein consisting of two chains of 21 and 30 amino acids. A schematic of the amino acid primary sequence is shown in Fig 1. Insulin forms complex oligomeric structures including, but not limited to, dimers and hexamers. Hexamers are typically stabilised with two zinc ion ligands. These oligomeric states are used to control the activity of insulin; it is the monomeric species which is most effective at reducing blood glucose, whereas the hexameric form is the most stable. Insulin analogues, specifically the fast-acting insulins, have been designed to prefer the monomeric and dimeric state, and thus increase the speed at which they enact a hypoglycaemic response. The mechanism for slow-acting insulin analogues is more complex but, in general, changes have been made to increase the propensity to form larger oligomeric states.

Numerous studies have examined different synthetic insulins using a range of different techniques [6,7]. However, in an attempt to understand further the structural characteristics of each synthetic insulin in relation to their relative clinical performance we have characterised, for the first time, all six clinically-available insulin analogues, as well as ‘native’ bovine, porcine and human insulins (shown in Fig 1), using gold-standard hydrodynamic methods with the most up-to-date analysis techniques.

## Materials and methods

### Insulins

Insulin analogues were sourced from commercial manufacturers as 10mL, 100U/mL (~3.5mg/mL) vials for injection except for detemir (IDet) and degludec (IDeg) which were obtained as 3mL vials for FlexTouch^®^ pens as a kind gift from the InDependent Diabetes Trust (IDDT). For categorisation, recombinant human, bovine and porcine insulins (IHr, IBov, IPor) are referred to as ‘Native insulins’, aspart, glulisine and lispro (IAsp, IGlu, ILis) are ‘Fast analogues’ and glargine, detemir and degludec (IGla, IDet, IDeg) are ‘Slow analogues’. Samples remained as their original preparations i,e, including all excipients present in the vial.

IHr, IAsp, IDet and IDeg were purchased from Novo Nordisk under the trade names ActRapid^®^, NovoRapid^®^ (a.k.a. NovoLog^®^ in USA), Levemir^®^ and Tresiba^®^ respectively. IHr and IAsp arrived as 100U/mL insulin, or 3.5mg/mL. IDeg was 3.6mg/mL, IDet was 14.2mg/mL, but both were the same potency/dose (100U/mL).

IGlu and IGla were purchased from Sanofi Aventis under the trade names Apidra^®^ and Lantus^®^, respectively. IBov and IPor were from Wockhardt, trade names Hypurin^®^ Bovine neutral and Hypurin^®^ Porcine neutral. ILis was purchased from Eli Lilly under the trade name Humulog^®^.

All insulins were stored refrigerated at 4–8°C and allowed to equilibrate to room temperature directly before analysis. Samples were analysed before their use-by date.

### Sequence properties

Sequences were inputted into SEDNTERP [8] to estimate partial specific volume ($\bar{v}$), hydration (δ) and isoelectric points (pI). Values are shown in supplemental data (S1 Table).

### Density and viscosity measurements

Solution density was measured using an Anton Paar DMA5000 oscillating capillary density meter (Anton Paar GmbH, Graz, Austria). Solution viscosity was measured using an Anton Paar AMVn rolling ball viscometer (6 d.mm silanised capillary, 5 d.mm steel ball). All measurements were performed at 20.00°C, controlled to within ±0.005°C, and are shown in S1 Table.

### Dynamic light scattering

Hydrodynamic radii were measured using Dynamic Light Scattering (DLS) in a Malvern Zetasizer NanoZS DLS (Malvern Instruments, Malvern, UK) at high scattering angle (173°) to minimise the effects of dust and supramolecular contamination (NIBS, 173°). Dynamic viscosity, required to convert diffusion coefficients to hydrodynamic radius (via the Stokes-Einstein equation), was taken from AMVn measurements. Plastic disposable cuvettes were capped and allowed to temperature equilibrate to (20.0±0.05)°C. Three replicates were made of each. Data were acquired using Zetasizer Software v6.22 (Malvern), based on the method of Provencher [9]. Results are shown in supplemental data (S2 Table, S1 Fig).

### Size exclusion chromatography coupled to multi angle light scattering (SEC-MALS)

Molar mass distributions were measured using SEC-MALS [10]. A Shimadzu (Kyoto, Japan) HPLC system was used in this investigation comprising a DGU-20A 3R degasser, LC-20AD pump, SIL-20A HT autosampler, CTO-20A column oven and CBM-20A communications module. The system was equilibrated with three different mobile phases, including two physiological buffers: PBS (10mmol PO_4_^3-^, 137mmol NaCl, 2.7mmol KCl, 0.35% cyclohexanol, 0.02% NaN_3_, pH measured at 7.4), and TRIS (10mmol TRIS, 140mmol NaCl, 0.35% cyclohexanol, 0.02% NaN_3_, pH adjusted to 7.7 with 1mol HCl); and a neutral, unbuffered Nitrate solution (100mmol NaNO_3_, 0.35% cyclohexanol, 0.02% NaN_3_). No zinc ions were added to any of the mobile phases. Mobile phases were chosen to represent physiological buffering systems, in which most of the insulins are already solvated, as well as an unbuffered, control system. 100μL of insulin samples (unmodified from their injectable drug formulations) were auto-injected into the mobile phase with a flowrate of 0.5mL/min. This size exclusion chromatography (SEC) column (30cm G3000PW_XL_, with a 12 μm pore TSK PW_XL_ guard column) was coupled to an 8-angle DAWN^®^ HELEOS^®^ II Multi-Angle Light Scattering (MALS) detector (Wyatt Technology, Santa Barbara, CA, USA), using a laser wavelength of 663.4nm, and Optilab^®^ T-rEX^®^ Differential Refractive Index Detector (Wyatt Technology) with a laser wavelength of 658nm. Data were acquired and analysed using ASTRA software v6.1.7.15 (Wyatt Technology). Elution plots from light scattering are presented in the main paper and differential refractive index shown in S3 Fig.

### Sedimentation velocity

Sedimentation coefficients and distributions were measured using sedimentation velocity analytical ultracentrifugation (SV-AUC), carried out using a Beckman Optima XL-I analytical ultracentrifuge (Beckman, Palo Alto, USA). Analytical ultracentrifuge cells were 2-channel (for solution and reference solvent) comprising 12mm aluminium-epoxy resin centrepieces and sapphire windows. 400μL of insulin preparations, along with a PBS buffer reference sample, were centrifuged at 45k rev/min (~155k *g*) at (20.0±0.1)°C and Rayleigh Interference scans taken every two minutes to measure their rates of sedimentation.

Scans were analysed using the *c*(*s*) vs. *s* algorithm in SEDFIT [11] v14.6e which is capable of yielding sedimentation coefficient distributions from individual scan sets and provide an estimate for the molar mass using a weight-average frictional ratio (*f/f*_*0*_). 100 scans were loaded to represent near-complete sedimentation, in approximately the first 10 hours of centrifugation. Sedimentation coefficients, s, were corrected to standard conditions, namely the density and viscosity of water at 20.0°C to yield s_20,w_ values.

### Sedimentation equilibrium

Sedimentation equilibrium AUC (AUC-SE) was performed on the same instrumentation and in the same cells as for AUC-SV (see above). 120μL of insulin preparations were centrifuged at (20.0±0.1)°C at a range of rotor speeds between 10k rev/min (~7.5k *g*) and 25k rev/min (~48k *g*). Equilibrium solute distributions were recorded using Rayleigh interference optics. Scans were analysed using SEDFIT-MSTAR [12], MULTISIG/MULTISIG-RADIUS [13] and INVEQ [14].

SEDFIT-MSTAR was used to measure the apparent weight-average for the whole distribution, M_w,app_, by the calculation of the M*(*r*) function:

$$M^{*}(r)=\frac{\left(c_{r}-c_{a}\right)}{kc_{a}(r^{2}-a^{2})+2k\int_{a}^{r}r(c_{r}-c_{a})dr}$$ (1)

where c is the concentration, r is the radial position, a is the meniscus radial position and k is a constant related to rotor speed, temperature and buoyancy. The M* function, extrapolated to the cell base b, is an approximation for the apparent, weight-average molar mass. Relative radius (r^2^-a^2^)/(b^2^-a^2^) was used to standardise the extrapolation, meaning a = 0 and b = 1. SEDFIT-MSTAR also yielded estimates of the point or local weight average molecular weights M_w_(r) as a function of local concentration c(r) in the cell. Values were also checked by the hinge-point analysis method which yields the M_w_(r) value at the point in the cell where c(r) = the cell loading concentration. SEDFIT-MSTAR also yielded estimates for the molar mass distribution c(M) vs M. For IDet, two speed analysis was used to extrapolate to a single point and provide information on a multi-component system. Otherwise, only one, averaged, scan was used for MSTAR analysis.

The MULTISIG algorithm was also applied to the sedimentation equilibrium distributions and was used to assess the distribution of molar mass. It was performed with 20 repeat fits at the hinge point and averaged to yield molar mass distributions, namely f(*M*) vs. M and the corresponding, number, weight, z-average molar masses for the distribution. For MULTISIG-RADIUS, the baseline was fixed to the previously fitted value from MULTISIG and f(*M*) vs. M was measured over the entire cell at 20 radial positions. The MULTISIG routine is capable of yielding multi-component/heterogeneous distributions although assumes thermodynamic non-ideality.

Although non-ideality effects are usually negligible when dealing with small molecules such as insulin–and the small concentration usually needed (<0.5mg/mL), because of the high concentrations being used in this study, non-ideality was probed using a variation of the INVEQ algorithm, which provides an estimate for the second thermodynamic virial coefficient *B* (mL.mol.g^-2^), or this value multiplied by the molar mass, M, *BM* (mL/g). The factor (1+2BMc), where c is the concentration (in g/mL) represents the factor by which the apparent molar mass, M_app_ measured at concentration c underestimates the true mass M. To a first approximation:

$$\frac{1}{M_{app}(c)}=\left(\frac{1}{M}\right)(1+2BMc)$$ (2)

## Results

Clinically available insulin analogues, using excipient content, solution attributes (density, viscosity), sedimentation behaviour (velocity and equilibrium) and static light scattering properties, were assessed in order to obtain stoichiometric and behavioural properties.

### Stoichiometry and structure

#### Solution properties

S1 Table shows the measured density and viscosity of the insulin preparations, as well as estimated parameters from SEDNTERP [15].

#### Dynamic light scattering

S2 Table summarises the hydrodynamic radius information obtained through dynamic light scattering (DLS). The distribution is also shown in S1 Fig. Typical hydrodynamic radii were 2–3nm, with smaller radii obtained for IGla and IDeg. Differences in radii cannot be reliably correlated to stoichiometry, but do provide a general trend. Peak volume percentages were all 100%. Since DLS is more sensitive to larger species than small, these findings strongly suggest no aggregates were present in any of the formulations.

#### Sedimentation velocity

**Fig 2:**
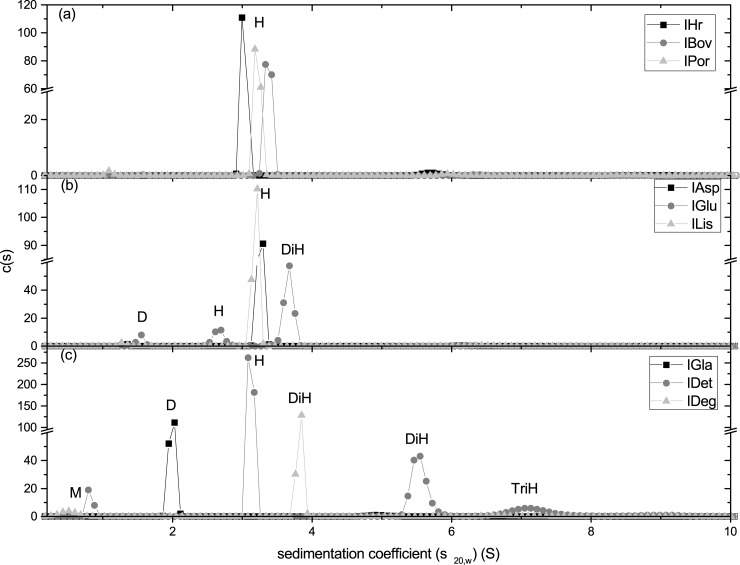
c(s) vs. sedimentation coefficient distributions measured using AUC-SV at 45k rpm. (a) Native insulins; (b) Rapid-acting analogues; (c) Slow-acting analogues. Monomers (M), Dimers (D), Hexamers (H), and Di and Tri Hexamers (Di/TriH) were identified by molar mass calculated through the sedimentation coefficient and the frictional ratio.

Scans were analysed using a c(s) fitting algorithm. Raw data (100 scans per sample) are shown in S2 Fig. along with superimposed fits as well as residual plots, Weight average sedimentation coefficients are shown in Table 1, obtained from distributions shown in Fig 2. Native insulins shared a similar sedimentation coefficient at 3S, however there is evidence of a small amount of sedimenting material at 5.5S for IHr (3% of distribution), which also showed a slightly slower sedimentation coefficient than IBov and IPor. No species were observed faster than 10S, and all macromolecular material was accounted for in the areas-under-the-curves, therefore no aggregation was present in these samples.

**Table 1:** Hydrodynamic parameters measured using AUC-SV and AUC-SE. Sedimentation coefficients are weight-averaged in the cases of multi-species distributions. IGlu and IDet yielded fits with BM<0. All insulins were measured at ~3.5mg/mL, except for IDet which was measured at 14.2mg/mL.

Insulin	sedimentation coefficient (s_20,w_, S)	Second virial coefficient (BM, mL/g)
IHr	3.0±0.1	20.6±0.3
IBov	3.4±0.1	2.89±0.23
IPor	3.2±0.1	5.17±0.22
IAsp	3.3±0.1	7.78±0.29
IGlu	3.4±0.1	N/D
ILis	3.2±0.1	1.65±0.05
IGla	2.0±0.1	26.5±0.2
IDet	3.8±0.1	N/D
IDeg	3.5±0.1	97.0±1.0

Fast-acting analogues shared similar behaviour to the native insulins, apart from IGlu which sedimented in three discrete peaks at 1.5 (8%), 2.7 (19%) and 3.8S (74%). Areas-under-the-curves, indicating the concentration of the macromolecular components, were consistent with 3.5mg/mL for all three analogues. This information, combined with a lack of species beyond 10S, suggest no aggregate was present in any of the native insulins.

Slow-acting analogues showed very different behaviour to native insulins and fast-acting analogues. IGla yielded a slower sedimenting peak at 2S. IDet sedimented at 3S (66%), but also showed peaks at 0.8 (4%), 5.5 (21%), and 7S (8%). This indicates a multi-hexameric conformation (1x hexamer, 2x hexamer, 3x hexamer) and potentially the insulin monomer at 0.8S. IDeg sedimented with a main peak at 3.8S (90%) and a smaller peak at 0.5S (8%).

It should be noted that these samples were analysed at single concentration, at 3,5mg/mL (or 14.2mg/mL for IDet) and therefore may show a degree of non-ideality unaccounted-for by the c(s) algorithm. Therefore these sedimentation coefficients are likely to be underestimates of the true sedimentation coefficient. However, non-ideality was measured using AUC-SE. These values, shown as the second virial coefficient *BM*, are shown in Table 1.

#### Sedimentation equilibrium

**Fig 3:**
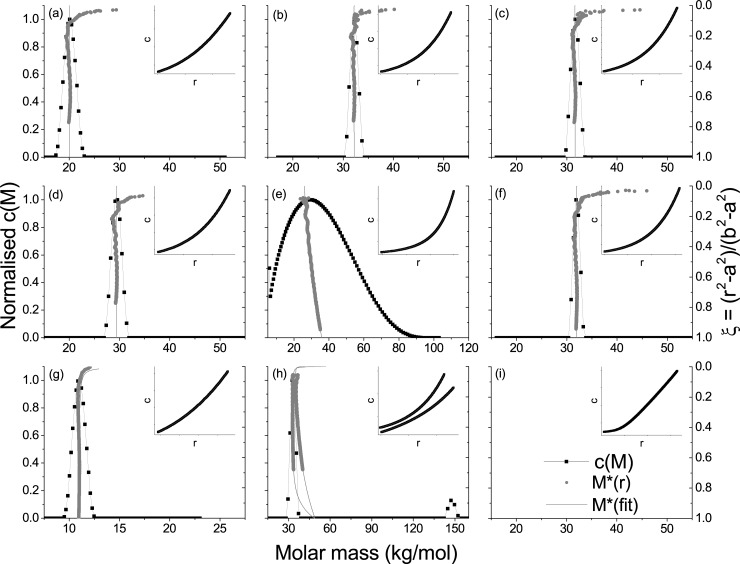
c(M) vs. molar mass distributions measured using SEDFIT-MSTAR from AUC-SE data, with superimposed M*(r) extrapolations to the cell base (relative radius = 1), and c(M) fits over raw AUC-SE data in insets. (a) IHr; (b) IBov; (c) IPor; (d) IAsp; (e) IGlu; (f) ILis; (g) IGla; (h) IDet; (i) IDeg. SEDFIT-MSTAR was unable to adequately fit data from IDeg.

**Fig 4:**
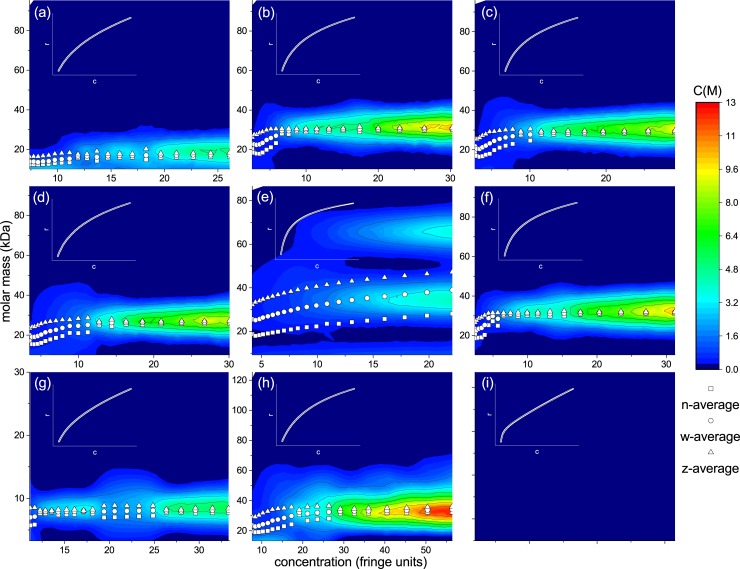
Molar mass distributions measured against concentration using MULTISIG-RADIUS from AUC-SE data, with superimposed n-, w- and z-average molar masses. Insets are fits from INVEQ analysis. (a) IHr; (b) IBov; (c) IPor; (d) IAsp; (e) IGlu; (f) ILis; (g) IGla; (h) IDet; (i) IDeg. MULTISIG-RADIUS was unable to adequately fit data from IDeg, but INVEQ was able.

From SEDFIT-MSTAR (Fig 3), native insulins all showed monodisperse systems at 20, 32 and 31 kg/mol for IHr, IBov and IPor, respectively. These molar masses are consistent with the approximate value for hexamer (36 kg/mol), consistent with findings from AUC-SV. However, the apparent mass of IHr was smaller than the IBov and IPor, which may be an indication of non-ideal behaviour. This was confirmed with INVEQ, where the second virial coefficient is nearly ten times higher than IBov (see Table 1). The distributions from SEDFIT-MSTAR were confirmed with MULTISIG-RADIUS (Fig 4). IHr yielded ~20 kg/mol and ~30 kg/mol for IBov and IPor. A small decrease in number-, weight- and z-averages were observed closer to the cell meniscus (low concentration end) going down to 20 kg/mol with a sigmoidal pattern, potentially indicating a self-interacting system. Second virial coefficients (BM) for the animal insulins fitted to 3–5mL/g, a typical value for proteins at this concentration, however IHr fitted a BM with a ten-fold higher value. This suggests a higher degree of non-ideality is present.

SEDFIT-MSTAR yielded similar distributions at 29 and 31 kg/mol for IAsp and ILis, respectively. However, IGlu yielded a very broad, polydisperse distribution between 10 and 90 kg/mol. The weight average is indicated to be 36 kg/mol from the M* extrapolation to the cell base. These results were replicated with MULTISIG-RADIUS, except for IGlu which was resolved into three consistent peaks throughout the concentration range at ~10, ~30 and ~65 kg/mol (dimer, hexamer, di-hexamer). This is a more resolved distribution than in Fig 3E yielded by SEDFIT-MSTAR, and is consistent with findings from AUC-SV (Fig 2B). This heterogeneity proved a challenge for analysis using INVEQ, yielding a BM<0, which is potentially an indication of self-association but may be an artefact of heterogeneous material on the fitting algorithm. However, IAsp and ILis had very low second virial coefficients.

Slow acting analogue IGla yielded a monodisperse, but smaller, distribution at 11 kg/mol. This is indicative of a dimer conformation. The multi-speed analysis feature in SEDFIT-MSTAR was used on two different rotor speeds for IDet to obtain a bimodal distribution. The weight average extrapolates to 49 kg/mol, whereas two peaks are present at 30 (hexamer) and 150 kg/mol (multi-hexamer).

IGla showed a single peak across the concentration range at 6000 g/mol using MULTISIG-RADIUS (smaller than obtained for SEDFIT-MSTAR). IDet only resolved one peak at ~30 kg/mol, inconsistent with results from SEDFIT-MSTAR and AUC-SV. However, AUC-SE is a low-resolution technique for distributions and it is therefore not surprising that a parsimonious model was fitted for this dataset to only show one peak. INVEQ was used to analyse the IDeg system, which yielded a weight-average molar mass of 33.1 kg/mol.

### Excipients and function

#### Excipients present in preparations

The list of excipients and concentrations for native insulins, along with fast and slow acting analogues, taken from patient information sheets and other relevant materials, are shown in S3 Table. Zinc was present in all but three preparations: IBov, IPor and IGlu. Phenol and/or m-cresol were present in all preparations, typically at ~3mg/mL where known. IGlu was the only insulin preparation not to contain glycerol, which was typically present at 16mg/mL. Buffer salts were not present in IHr, IGla or IDeg, and insulins which did have buffering salts were inconsistent with their concentrations and types, although mostly contained TRIS or sodium phosphate. Polysorbate 20, also known as Tween® 20, was present in Sanofi insulins IGlu and IGla.

#### Size-exclusion chromatography

**Fig 5:**
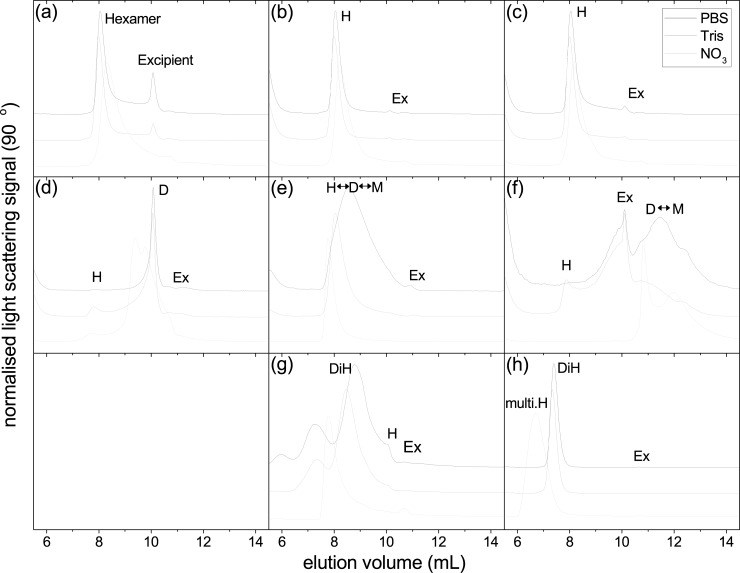
Elution from SEC plots of insulin and analogues. Black line represents PBS as the mobile phase, grey line represents TRIS. (a) IHr; (b) IBov; (c) IPor; (d) IAsp; (e) IGlu; (f) ILis; (g) IDet; (h) IDeg. IGla was not injected due to pI/pH incompatibilities. Monomers (M), Dimers (D), Hexamers (H), Dihexamers (DiH), multihexamers (multiH) and Excipients (Ex) were identified by molar mass.

Native insulins eluted at 16 minutes/8mL (MALS confirmed this peak to be ~30–40 kg/mol, or hexameric conformation) with small amounts of material eluting at 10mL (100–200 g/mol, likely to be an excipient peak, e.g. glycerol). Although the light scattering signal was high, very little signal was observed from differential refractive index (S3 Fig) suggesting there may be very large aggregates present, which would usually scatter large amounts of light, but in very small quantities as to not impact the refractive index. There was no apparent difference between PBS, TRIS and Nitrate for native insulins. The elution profiles are shown in Fig 5 and weight-average molar masses summarised in Table 2.

**Table 2:** Weight-average molar mass estimates from AUC-SV, AUC-SE and SEC-MALS results. Estimates uncorrected for non-ideality are labelled ‘apparent’ (_app_). All AUC-SV estimates were made on data from 45k rpm and based upon estimates of the frictional ratio fitted from boundary spread, and are weight averaged in the case of multiple sedimenting species. AUC-SE analysis was made at the indicated rotor speeds in parentheses (k rpm). SEC-MALS estimates are shown as consensus values from PBS and TRIS mobile phases. IDeg was unanalysable using MSTAR and MULTISIG. IGla was not assayed using SEC-MALS due to pI/pH incompatibilities.

Insulin	AUC-SV M_w,app_ kg/mol	AUC-SE MSTAR M_w,app_ kg/mol (k rpm)	AUC-SE MULTISIG M_w,app_ kg/mol (k rpm)	AUC-SE INVEQ M_w_ kg/mol (k rpm)	SEC-MALS M_w,app_ kg/mol
IHr	40.3	20.1 (18)	16.9 (18)	26.8 (18)	30.0
IBov	36.0	32.2 (18)	30.1 (18)	33.3 (18)	31.9
IPor	38.1	31.6 (18)	28.8 (18)	33.5 (18)	31.5
IAsp	38.0	29.4 (18)	26.2 (18)	32.6 (18)	12.4
IGlu	29.8	35.9 (15)	32.9 (15)	44.7 (15)	24.0
ILis	36.9	31.9 (18)	31.3 (18)	33.3 (18)	10.4
IGla	21.7	8.1 (25)	8.1 (25)	14.8 (25)	N/D
IDet	60.4	49.2 (10,18)	33.5 (18)	33.8 (18)	60.2
IDeg	92.2	N/D	N/D	33.1 (25)	66.0

Fast-acting analogues did not elute as consistently as the native insulins. Only a small amount of IAsp material eluted at 8mL, with the majority eluting at the excipient peak (10 minutes). IGlu eluted with a broad distribution between 8 and 10mL (this was broader for PBS than for TRIS or Nitrate). ILis eluted much later (12mL) than the excipient peak with a broad distribution eluting as late as 13mL. There was also a difference between elutions obtained in TRIS compared to PBS and Nitrate for ILis.

IDeg eluted at 7.5mL (MALS indicated this peak to be 60–70 kg/mol) in PBS and TRIS, and 7mL for Nitrate (270 kg/mol) with no indication of an excipient peak at 10mL as seen in other samples. IDet eluted with multiple peaks between 6–10mL, with a shoulder peak observed at 10mL indicating the excipients. A small difference was observed between TRIS and PBS for IDet: in TRIS, the main peak eluted 0.5mL earlier and there is a smaller peak at 5.5mL, however Nitrate eluted at 8mL with a MALS confirmed molecular weight of 300 kg/mol.

## Discussion

### Analytical ultracentrifugation

Our results indicate that native animal insulins exist primarily in the hexameric form with a sedimentation coefficient of 3.0S and with 95% of the loading concentration. In 1931 Sjögren and Svedberg [16] investigated preparations of bovine insulin at pH ~7 by sedimentation velocity and equilibrium, and using a partial specific volume (measured by pycnometry) of 0.750mL/g. These researchers found sedimentation coefficients ranging from 3.21–3.41 x10^-13^ s_20_, and molar mass of 35.1 kg/mol. We now know that this is consistent with the hexameric conformation. Numerous other studies involving AUC were performed on animal insulins (bovine and porcine) since this seminal paper all homing in on the modern interpretation of the monomer-dimer-hexamer stoichiometry, with 5.8 kg/mol subunits [17–23], with the hexamer precisely stabilised and controlled with zinc ions [24–26] to a point where an excess causes aggregation [27]. Once protein expression from micro-organisms became possible, researchers turned their attention to human recombinant insulin, either corroborating the findings from animal insulin experiments [28–30] or researching insulin’s fibrillation properties [31]. This investigation builds on the work of these publications by using the most recent advances in AUC-SV and SE analysis.

Fast-acting analogues were also studied using AUC-SV and AUC-SE. IAsp and ILis were found to exist in a mostly hexameric state, whereas IGlu was in a complex dimer-hexamer-dihexameric state. IAsp and IGlu have only been studied using sedimentation velocity once before by Teska et al. [32], and there have never been any publications on the use of sedimentation equilibrium on either of these analogues. Teska et al. studied the effects of phenolic preservatives and zinc concentration on fast acting analogues, as well as the drug-formulation available to patients. They found similar c(s) profiles to this present investigation, including the three-peaked distribution from IGlu. ILis, the oldest marketed analogue available to patients, however, has been studied more extensively [29,33]. Richards et al. [33] also used sedimentation equilibrium, yielding differential, point-average, molar mass against concentration plots. No non-ideality was observed due to lower concentrations used in their study. Also, minimal polydispersity was observed as the molar mass remained consistent throughout the cell in the monomeric state (5.8 kg/mol). These findings were corroborated by Hinds et al. [30] who found the system to be entirely monomeric, using similar analysis techniques to Richards et al [33]. The latest findings from this investigation suggested that the system was mostly hexameric, however the results are from injectable drug formulations and would therefore be designed to keep the insulin in the most protective stoichiometry. It is also the first published account of insulins aspart and glulisine analysis using AUC-SE.

It is interesting to note that the study of insulins glargine and detemir using AUC (either SV or SE) have been limited or non-existent. IGla was reported by our group [3] as part of a dosage study involving the single and triple dose forms and similar results are observed in this present investigation. IDet showed interesting multi-hexameric behaviour in AUC-SV, but this was not present in AUC-SE; possibly due to the very high concentration involved (and therefore high signal from the main peak) as well as the low-resolving power from SE analysis.

Steensgaard et al. [34] using AUC-SV, SE as well as other complementary techniques, investigated and discovered multi-hexameric behaviour in IDeg. Steensgaard et al. posited that multihexamers combine to form long rods, or fibres, which aid in the action of retarding the release of efficacious monomers into the bloodstream. Although we did not find similar multi-hexameric behaviour in IDeg, we did observe dihexamers. In addition, we found a high degree of non-ideality, confirmed from a previous investigation [3] to be a result of a lack of buffering salts in the drug preparation. It is possible that the excipient composition in the product prevents the formation of these fibres.

### Size exclusion chromatography

This investigation employed SEC-MALS to assess both the quaternary structure, in the form of stoichiometry, as well as function, in relation to the absence of zinc ions. Eight of the nine insulins were injected into the column (IGla was incompatible with the mobile phases) and assessed in terms of elution time and measured molar mass. IHr, IBov and IPor all behaved in a similar fashion, eluting at the same time and existing in the hexameric state, despite having a mobile phase void of zinc ions. This leads to two opposing possibilities to be posited; first, that the lack of zinc ions allowed for diffusion of bound zinc from the complex, and that the intra-protein attraction was still strong enough to maintain the hexamer; second, the binding strength of the complex to the zinc ions was too strong to allow zinc to diffuse into the mobile phase. From this investigation it is unclear which of these two scenarios is true, however it is our belief that the second option is more likely.

A series of seminal papers by Brange et al. [35–39] employed size exclusion chromatography (SEC) to study the formation of covalently bound insulin dimers in native insulins and the effects of excipients and storage conditions. Among other findings, they suggested that the use of phenol and phenol-like compounds reduced the incidence of covalent dimer formation. This is the reason why these compounds were present in all formulations tested in this investigation, although Brange’s research showed that effectiveness was greatest with phenol, then m-cresol, then methyl paraben yet the insulin preparations contained mostly m-cresol. This is likely due to the higher toxicity of phenol compared to m-cresol. Findings from this investigation showed no evidence of covalent dimers, likely due to the presence of these phenolic preservatives.

Fast acting analogues behaved very differently from native insulins. IAsp and ILis eluted as primarily dimers and monomers, and IGlu eluted as a monomer-dimer-hexamer stoichiometry. In this respect, IGlu behaved in the most similar fashion to AUC experiments. The mobile phase also appears to have made a difference; nitrate buffer caused IGlu to form mostly hexamers, and ILis elutions were erratic between the three buffers. It is likely that the different cations, being of different size and charge density, acted upon the insulins in distinct ways, however the mode of action is not clear from this investigation.

Bakaysa et al. [29] employed static light scattering to confirm the molar mass obtained from AUC-SE for ILis, although this was not combined with a chromatographic column. They found the system to act in a similar, dimeric fashion, however in this investigation the two techniques yielded different molar masses. ILis showed a broad monomer-dimer distribution eluting the column much later, and confirmed with MALS. This is due to the presence of excipients and zinc ions in AUC-SE which would have diffused away from the bolus and caused the system to dissociate. The same phenomenon should have occurred for the native insulins if it were not for the fact that these fast acting analogues were designed to dissociate in this way, and is in agreement with the mode of action *in vivo*, where fast-acting analogues are injected subcutaneously and dissociate into the more effective monomer form faster than regular insulin. Similar conclusions were drawn by Gast et al., [40] who also studied the association/dissociation kinetics of these fast acting analogues.

IGla has been previously studied using DLS [41,42]. Both studies found IGla to exist in large aggregates in pH 7 due to the isoelectric point matching the buffer. In this investigation (S2 Table), the drug formulation showed IGla to exist as very small oligomers–although DLS is not sensitive enough to precisely indicate in which oligomeric state it exists, the results are consistent with the findings from AUC-SV that showed the presence of dimers (in that the particle size was significantly smaller than the hexameric insulins). Nakazawa et al. [41] also studied IDet using DLS, but is the only paper to publish on this method for this analogue. They found the hydrodynamic diameter of IDet to be larger than IHr, ILis and IGlu, consistent with findings from this investigation.

Havelund et al. [43] studied IDet, in the context of the phenomenon of albumin binding, using SEC. They used TRIS-buffered saline at 37°C in the presence and absence of 2mmol/L phenol, as opposed to this investigation studying all analogues in the presence of cyclohexanol, an equivalent compound, in three different buffering systems. Havelund et al. also found IDet to form a hexamer-dihexamer equilibrium but no higher-order multihexamers. However, these findings may be due to the concentration range used, as they injected 3.5mg/mL whereas 14mg/mL was used for this investigation, the concentration which is clinically applicable to this insulin.

## Conclusions

Nine clinically available and administered insulins and analogues have been examined using hydrodynamic and light scattering techniques. This is the first time all nine insulins have been assessed in this way, the first time that analytical ultracentrifugation has been employed on insulin detemir, and the first time that insulins aspart and glulisine have been assayed using a sedimentation equilibrium experiment in publication. Analytical ultracentrifugation revealed the quaternary structures of all nine insulins, revealing complex, but clinically important, stoichiometries. Size exclusion chromatography then showed the function of these analogues when zinc, the ligand critical to the structure and stability of the insulin hexamer, was removed from the mobile phase. These characteristics are important for future developments in insulin, and in developing new personalised treatments for diabetes.

## Supporting information

S1 Fig Distributions (volume percentage against hydrated radius) obtained through dynamic light scattering. (a) native insulins, (b) fast-acting analogues, (c) slow-acting analogues.
(EPS)

S2 Fig Raw data and superimposed fits from 100 scans taken over the course of sedimentation velocity experiments of all nine insulins. Residual plots (fit-raw) are shown underneath sedimentation profiles. (a) IHr, (b) IBov, (c) IPor, (d) IAsp, (e) IGlu, (f) ILis, (g) IGla, (h) IDet, (i) IDeg.
(TIF)

S3 Fig Elution profiles of insulins by three different mobile phases (PBS, TRIS and Nitrate) detected by differential refractive index. (a) IHr, (b) IBov, (c) IPor, (d) IAsp, (e) IGlu, (f) ILis, (g) IDet, (h) IDeg.
(EPS)

S1 Table SEDNTERP output and density/viscosity properties for insulin and analogue samples at 20°C. (DOCX)

S2 Table Results from dynamic light scattering of insulin and analogues. Measurements taken at an angle of 173° and at 20°C.
(DOCX)

S3 Table List of excipients found from patient safety information sheets, and related literature, for insulin and analogues (per millilitre of preparation). +, the excipient is mentioned but not quantified. -, not present or not mentioned. Trace, as presented in documentation.
(DOCX)
