# Supplementary figures and images for: Characterisation of insulin analogues therapeutically available to patients
Source: PLoS One. 2018 Mar 29;13(3):e0195010. doi: 10.1371/journal.pone.0195010 (PMC5875863; doi:10.1371/journal.pone.0195010)

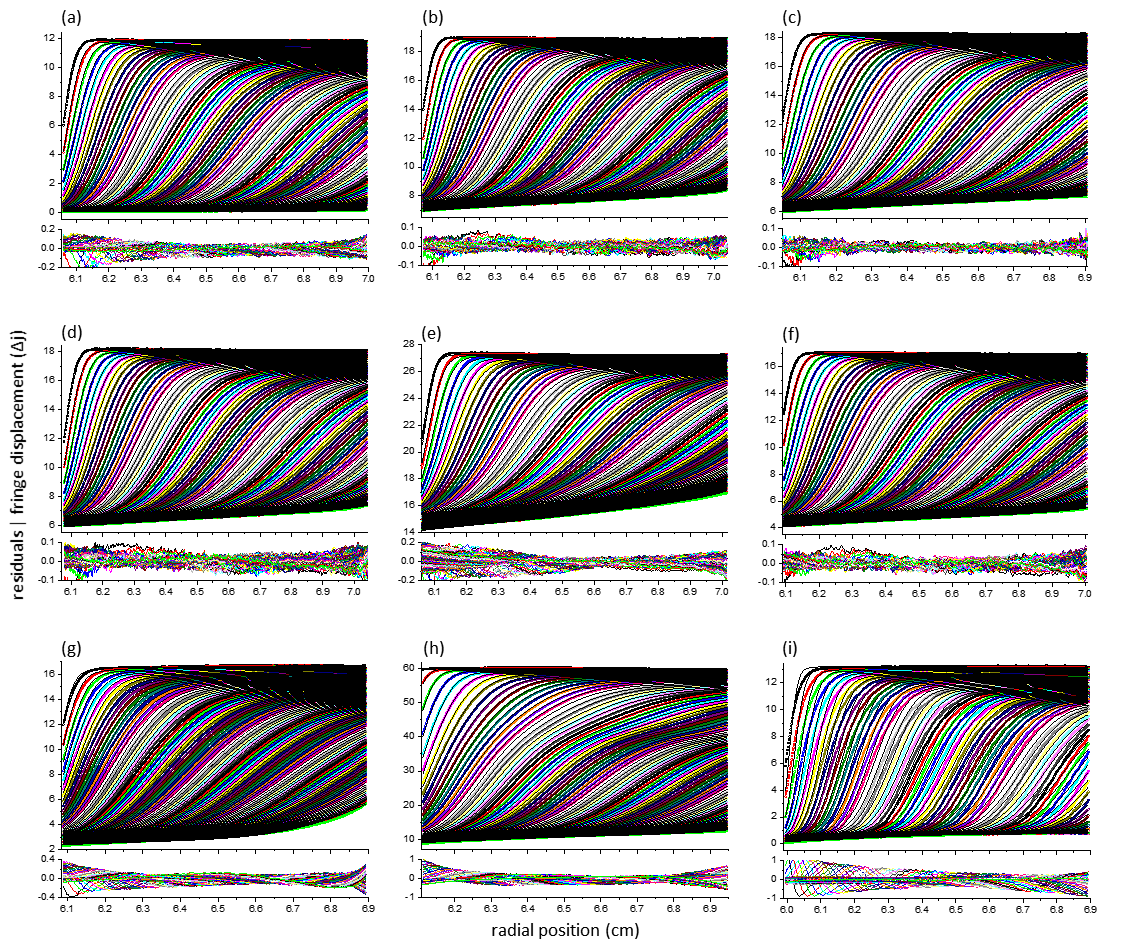

Supplement: S2 Fig — (a) IHr, (b) IBov, (c) IPor, (d) IAsp, (e) IGlu, (f) ILis, (g) IGla, (h) IDet, (i) IDeg. (TIF) [file pone.0195010.s002.tif]
