# Supplementary material for: Characterisation of insulin analogues therapeutically available to patients
Source: PLoS One. 2018 Mar 29;13(3):e0195010. doi: 10.1371/journal.pone.0195010 (PMC5875863; doi:10.1371/journal.pone.0195010)
Supplement: S1 Table — (DOCX) [file pone.0195010.s004.docx]

**S1 Table. SEDNTERP output and density/viscosity properties for insulin and analogue samples at 20^o^C.**

|  | **Molar mass ^a^ (Da)** | **^a^**  **(mL/g)** | **δ ^a^**  **(g/g)** | **pI ^a^** | **Extinction coefficient ^a^ (mL/mg.cm)** | **Solution density ^b^ (g/mL)** | **Solution viscosity ^c^ (mPa s)** |
| --- | --- | --- | --- | --- | --- | --- | --- |
| IHr | 5807.7 | 0.728 | 0.318 | 5.58 | 1.098 | 1.003474 | 1.090 |
| IBov | 5733.6 | 0.728 | 0.319 | 5.58 | 1.112 | 1.004558 | 1.146 |
| IPor | 5777.6 | 0.729 | 0.318 | 5.58 | 1.103 | 1.004580 | 1.094 |
| IAsp | 5825.6 | 0.725 | 0.326 | 5.13 | 1.094 | 1.004945 | 1.104 |
| IGlu | 5822.7 | 0.729 | 0.334 | 5.14 | 1.095 | 1.005552 | 1.064 |
| ILis | 5807.7 | 0.728 | 0.318 | 5.58 | 1.098 | 1.004598 | 1.106 |
| IGla | 6063.0 | 0.728 | 0.320 | 6.95 | 1.051 | 1.003799 | 1.107 |
| IDet | 5916.9 | 0.738 | 0.305^d^ | 5.58^d^ | 1.117^d^ | 1.008029 | 1.139 |
| IDeg | 6104.0 | 0.736 | 0.321^d^ | 5.14^d^ | 1.092^d^ | 1.004356 | 1.118 |

^a^ From SEDNTERP, monomer only, two chains, three disulphide bridges

^b^ From oscillating capillary densitometry

^c^ From rolling ball viscometry

^d^ Uncertain values due to fatty acid conjugations
