# Supplementary material for: Characterisation of insulin analogues therapeutically available to patients
Source: PLoS One. 2018 Mar 29;13(3):e0195010. doi: 10.1371/journal.pone.0195010 (PMC5875863; doi:10.1371/journal.pone.0195010)
Supplement: S2 Table — Measurements taken at an angle of 173° and at 20°C. (DOCX) [file pone.0195010.s005.docx]

**S2 Table. Results from dynamic light scattering of insulin and analogues.** Measurements taken at an angle of 173^o^ and at 20^o^C.

| **Insulin** | **Hydrated radius (nm)** | **Peak SD width (nm)** | **Diffusion coefficient (nm^2^/s)** | **Peak volume percentage (%)** |
| --- | --- | --- | --- | --- |
| IHr | 1.94 | 0.36 | 10.2 | 100 |
| IBov | 2.25 | 0.54 | 8.33 | 100 |
| IPor | 2.98 | 0.57 | 6.59 | 100 |
| IAsp | 2.45 | 0.40 | 7.94 | 100 |
| IGlu | 2.86 | 0.53 | 7.06 | 100 |
| ILis | 2.56 | 0.49 | 7.58 | 100 |
| IGla | 1.34 | 0.20 | 14.4 | 100 |
| IDet | 2.61 | 0.93 | 7.24 | 100 |
| IDeg | 1.21 | 0.23 | 15.9 | 100 |
