# Supplementary material for: Characterisation of insulin analogues therapeutically available to patients
Source: PLoS One. 2018 Mar 29;13(3):e0195010. doi: 10.1371/journal.pone.0195010 (PMC5875863; doi:10.1371/journal.pone.0195010)
Supplement: S3 Table — +, the excipient is mentioned but not quantified. -, not present or not mentioned. Trace, as presented in documentation. (DOCX) [file pone.0195010.s006.docx]

**S3 Table. List of excipients found from patient safety information sheets, and related literature, for insulin and analogues (per millilitre of preparation**). +, the excipient is mentioned but not quantified. -, not present or not mentioned. Trace, as presented in documentation.

| **Insulin** | **Protein**  **(mg)** | **Zinc**  **(μg)** | **Phenol**  **(mg)** | **m-Cresol**  **(mg)** | **Glycerol**  **(mg)** | **Buffer salts**  **(mg)** | **Other** |
| --- | --- | --- | --- | --- | --- | --- | --- |
| IHr | 3.50 | + | - | + | + | - | - |
| IBov | 3.42 | - | 0.6 | 1.6 | + | +^a^ | - |
| IPor | 3.45 | - | + | + | + | +^a^ | - |
| IAsp | 3.50 | 19.6 | 1.5 | 1.72 | 16 | 1.83^b^ | - |
| IGlu | 3.49 | - | - | 3.15 | - | 11^c^ | Polysorbate 20 |
| ILis | 3.50 | 19.7 | Trace | 3.15 | 16 | 1.88^d^ | - |
| IGla | 3.64 | 30 | - | 2.70 | 16 | - | Polysorbate 20 |
| IDet | 14.2 | 65.4 | 1.8 | 2.06 | 16 | 2.06^e^ | - |
| IDeg | 3.66 | 32.7 | 1.5 | 1.72 | 19.6 | - | - |

^a^ Sodium phosphate

^b^ 1.25mg/mL dibasic sodium phosphate dihydrate, 0.58mg/mL sodium chloride

^c^ 6mg/mL Trisaminomethane, 5mg/mL sodium chloride

^d^ 1.88mg/mL dibasic sodium phosphate

^e^ 0.89mg/mL dibasic sodium phosphate dihydrate, 1.17mg/mL sodium chloride
